# Single Nucleotide Polymorphisms in the 3′UTR of VPAC-1 Cooperate in Modulating Gene Expression and Impact Differently on the Interaction with miR525-5p

**DOI:** 10.1371/journal.pone.0112646

**Published:** 2014-11-12

**Authors:** Fabiana Paladini, Nicla Porciello, Giorgio Camilli, Sinem Tuncer, Elisa Cocco, Maria Teresa Fiorillo, Rosa Sorrentino

**Affiliations:** Department of Biology and Biotechnology “Charles Darwin”, Sapienza University of Rome, Rome, Italy; Nanjing Medical University, China

## Abstract

Complex immune and neurodegenerative disorders are the result of multiple interactions between common genetic variations having, individually, a weak effect on the disease susceptibility or resistance. Interestingly, some genes have been found to be associated with more than one disease although not necessarily the same SNPs are involved. In this context, single nucleotide polymorphisms in the 3′UTR region of type 1 receptor (VPAC-1) for vasoactive intestinal peptide (VIP) have been reported to be associated with some immune-mediated as well as with neurodegenerative diseases such as Alzheimer's Disease (AD). Here, we demonstrate that variations at the 3′UTR of the VPAC-1 gene act synergistically to affect the expression of the luciferase as well as of the GFP reporter genes expressed in HEK293T cells. Moreover, the miRNA 525-5p, previously shown by us to target the 3′UTR of VPAC-1, is more efficient in decreasing GFP expression when co-expressed with constructs carrying the allele C at rs896 (p<10^-3^) suggesting that this miRNA regulates VPAC-1 expression at different levels depending on rs896 polymorphism and thus adding complexity to the network of disease susceptibility.

## Introduction

VIP is a 28mer pleiotropic neuropeptide belonging to a family of structurally related neuropeptides and hormones mainly involved in the maintenance of neuroendocrine-immune system communication and released by both neurons and immune cells. It acts through specific receptors, VPAC-1 and VPAC-2, having different tissue distribution but similar affinities for VIP [1]. These receptors belong to the group II of G-protein-coupled receptors, the secretin receptor family, and couple principally to the adenylate cyclase (AC)-cyclic AMP pathway [2]. VIP, when released in the lymphoid organs by nerve stimuli and by activated immune cells, modulates the function of inflammatory cells through its receptors, thus contributing to the crosstalk between neural and immune systems.

During innate and adaptive immune responses, VIP behaves as a negative tuner of inflammation by suppressing the production of pro-inflammatory cytokines and chemokines from macrophages, microglia and dendritic cells [3]. Besides, VIP decreases the expression of co-stimulatory molecules, such as CD80 and CD86 on the antigen presenting cells, and, consequently, affects the activation of antigen-specific CD4+ Th1 cells while promoting Th2-type responses [3]. More recently, VIP has been shown to induce the emergence of regulatory T cells with suppressive activity against autoreactive effector T cells [4]. Moreover, VIP is involved in the maintenance of gastrointestinal homeostasis, both as a neurotransmitter and neurotrophic factor but also as an inducer of the of submucosal glands secretion [5].

Recently, the therapeutic effectiveness of VIP in several animal models of inflammatory/autoimmune diseases, such as inflammatory bowel disease, collagen-induced arthritis, experimental autoimmune encephalomyelitis, endotoxic shock, Parkinson's disease, Sjogren's and Crohn's diseases has been proven [6]–[13]. Accordingly, VIP and its analogues are in the spotlight as very promising candidates for treating acute and chronic inflammation [14].

The VIP receptor 1 (VPAC-1) appears to be the principle vehicle through which VIP exerts its regulatory function. VPAC-1 is constitutively expressed by unstimulated CD4+CD8+ double positive thymocytes, by peripheral lymphocytes, monocytes/macrophages and neutrophils [15]–[18]. Interestingly, VPAC-1 has been reported to be down-modulated in cells of the immune system after activation, arguing for a role of VIP/VPAC-1 signaling to keep in check the inflammatory response [19]. Monocytes, in particular, express detectable levels of VPAC-1 [20] which rapidly decrease following a powerful inflammatory stimulus such as that by the endotoxin LPS [21]. In this regard, we have shown that the presence of specific allelic variants in the 3′UTR region associate with different kinetics of VPAC-1 down-modulation [22]. Interestingly, we found that, in monocytes, this is mediated by the action of the microRNA 525-5p whose expression was quickly up-regulated following LPS stimulation. Accordingly, a putative target sequence of the microRNA 525-5p was identified in the VPAC-1 3′UTR in proximity of the SNP rs896. It maps within a stretch of AT (TTTTT/CAAA) where the T/C substitution could modify the secondary structure of the messenger RNA. However, the experimental settings used in that study did not allow the detection of any preferential miR-525-5p targeting of the rs896 sequence. Nevertheless, the experiments performed in U937 cell line and primary monocytes confirmed that VPAC-1 mRNA is one of the targets of miR-525-5p [21]. The modulation by miRNAs might be part of the complex network that makes VPAC-1 polymorphisms associated with some complex autoimmune and neurodegenerative diseases [23]–[27].

In this context, a recent Genome Wide Association Study (GWAS) has provided evidence that the genetic variant rs897 in 3′UTR of VPAC-1, mapping 31 bases from rs896, is strongly associated with Alzheimer's disease (AD), especially with the late-onset form (LOAD) that has a complex multifactorial background [28]. Moreover, it has been very recently shown that the neurogenic effect of microglia on hippocampal precursor cells is mediated by VPAC1 expression [29]. Given the relevance of VPAC-1 in regulating the neuro-immune and the inflammatory networks and its association with some severe diseases, we decided to investigate in more details the effect of the four different rs896 and rs9677 SNP extended haplotype combinations (C-C; C-T; T-C; T-T) on gene expression as well as on the modulation exerted by miR-525-5p. The results indicate that variations at 3′UTR act synergistically to influence the basal expression of different reporter genes and that the effect of miR-525-5p is significantly increased in the presence of a C at rs896.

## Materials and Methods

### Ethics statement

This study has been approved by the Ethics Committee of Policlinico Umberto I, Sapienza University of Rome (N°1815-January 29, 2010). All participants involved in this study gave their written informed consent.

### PCR amplification and molecular cloning

The entire 3′UTR region (1131 bp) of VPAC-1 (Genebank accession number NM_004624) was amplified from the human genomic DNA extracted from blood of healthy donors using Nucleospin Blood kit (Macherey-Nagel, Bethlehem, PA, USA) [21]. The typing of SNPs rs896 (C/T) and rs9677 (C/T) was performed by specific kits optimized for allelic discrimination through real time PCR by the use of the TaqMan probes specific for each variant (TaqMan SNP Genotyping Assay, C_3056870 and C_3056885, respectively; Applied Biosystems, Foster City, CA, USA). We proceeded to amplify and clone the four possible rs896-rs9677 haplotype combinations of the 3′UTR of VPAC-1, starting from the DNA whose sequence differences are reported in Table 1. The primers used are the followings:

**Table 1:** 3′UTR VPAC-1 haplotypes cloned in pGL3, psi-Check2 and pEGFP-N1gene expression vectors.

rs763625	rs897	rs895	rs896	rs342511	rs14380	rs8913	rs9677
A	C	C	**C**	A	T	A	**C**
C	C	C	**C**	A	A	A	**T**
A	T	T	**T**	G	A	G	**C**
A	T	T	**T**	G	A	G	**T**

for pGL3 and psi-Check-2 vectors:

3′UTR_FRW: 5′-GCGCGC-TCTAGA-GACACTCCTAGAGAACGCAG-3′

3′UTR_REV:5′-GCGCGC-TCTAGA-CTCCTATCCAGATGATACATGAG-3′

for pEGFP-N1:

3′UTR_FRW: 5′-GCGGCCGCG-AAGGAAAAAA-ACACTCCTAGAGAACGCAG-3′

3′UTR_REV: 5′-GCGGCCGC-AAGGAAAAAA-TCCAAGCCAACATTTATTGT-3′

All primers were designed using the software “perlprimer.sourceforge.net”. The amplified DNA fragments were cloned into the pGL3, psiCHECK-2 and pEGFP-N1 vectors (Promega, Madison, WI, USA) at the proper restriction sites. Plasmids carrying each of the four haplotypes were checked by restriction analysis and DNA sequencing.

### Transfection and dual luciferase reporter assay

HEK293T (ATCC cat. CRL-11268) cells were seeded in 24-well plates (15×10^4^/well) in complete Dulbecco's modified Eagle's medium (DMEM) containing 10% fetal bovine serum (FBS) and transfected after 24 hours with Lipofectamine 2000 (all purchased from Gibco, Invitrogen, Carlsbad, CA, USA) according to the instructions provided by the manufacturer. A total of 305 ng plasmid DNA/well was transfected, including 300 ng pGL3 recombinant construct plasmid or empty vector control and 5 ng pRL-TK (Promega) containing the Renilla luciferase gene. For psiCHECK-2 analysis we used 300 ng of recombinant constructs or empty vector. Transfections were performed in triplicate. The cells were lysed in a standard lysis buffer (Promega) for 30 min at room temperature (RT) and the cell lysates were assayed for both Firefly and Renilla luciferase activity using the Dual-Luciferase Reporter assay kit (Promega). Fluorescence was detected using a microtiter plate reader (Victor2) and the software Wallac 1420 (PerkinElmer, Challenger Drive Alameda, CA, USA). Relative luciferase activity was determined by normalizing the Firefly luciferase activity (F value) against the Renilla luciferase activity (R value). Each experiment was performed in triplicate. The F/R value was calculated to obtain an average value. Values were then normalized to the average value of the empty vector (no insert) within the same experiment to yield the vector-normalized ratio, or Relative luciferase Activity. For pEGFP-N1 constructs examination, HEK293T cells were transfected with 500 ng of empty vector or plasmid construct carrying one of the four haplotypes and assessed by flow cytometric analysis (BD FACSCalibur flow cytometer, BD Bioscience, Qume Drive San Jose, CA, USA). The Expression Index was calculated as mean fluorescence intensity, corrected for non-specific fluorescence in combination with the percentage of positive cells (% pos) and expressed as fraction of the value obtain from cells transfected with empty pEGFP-N1.

### Transfection and microRNA525-5p analysis

Double-stranded RNAs that mimic mature miRNAs, miR-525-5p and miRNA negative control were obtained from Dharmacon (Lafayette, USA). The transfection of HEK293T cells [21] was optimized utilizing Lipofectamine 2000 Reagent (Invitrogen) according to the manufacturer's instructions. Cells were seeded in 24 well plates and transfected with 500 ng of each pEGFP-N1 construct carrying one of the four haplotypes and 40 picomoles of the appropriate mimic miRNAs. 24h after transfection, cells were analysed by flow cytometry analysis. The Expression Index was calculated as mean fluorescence intensity (MFI), corrected for non-specific fluorescence (Fwt autofluorescence of wild type cells) in combination with the percentage of positive cells (%pos) and expressed as fraction of the value obtained from cells transfected with the control, according to the following formula: 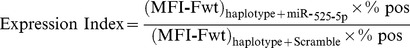



### Statistical analysis

Differences in the level of expression were analyzed by non-parametric statistical test Kruskal-Wallis and Dunn's multiple comparative post-test, using GraphPad Instat software. p values <0.05 were considered statistically significant.

## Results

Haplotype combinations of SNPs rs896 and rs9677 in the 3′UTR of VPAC-1 differ significantly in modulating the luciferase and GFP gene expression in HEK293T cell transfectants.

**Figure 1:**
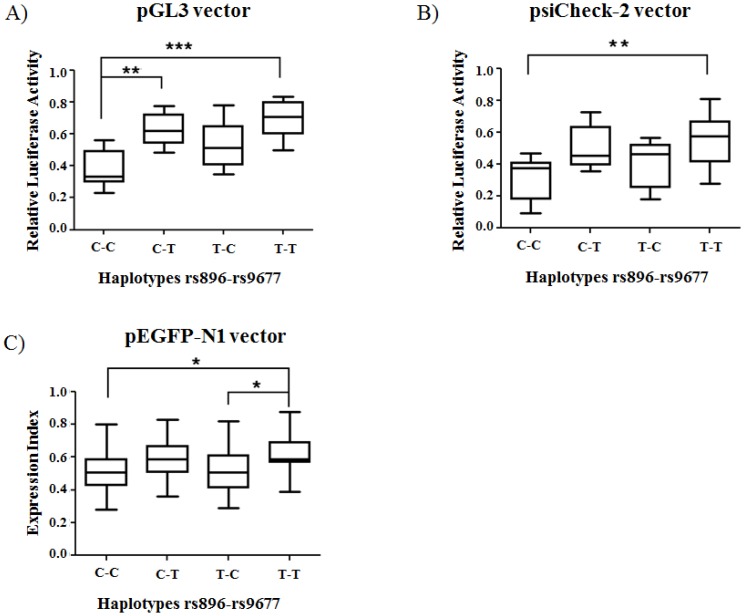
VPAC1 3′UTR haplotypes differently modulate the expression of reporter genes. (A) Box and whiskers plots of the relative luciferase expression from pGL3 reporter constructs transfected into HEK293T cells. Cells were transfected with pGL3 luciferase reporter plasmid carrying the full-length 3′UTR VPAC-1 (1131bp) containing each one of the four rs896-rs9677 haplotypes (C-C, C-T, T-C and T-T). The pRL-TK, a plasmid expressing Renilla Luciferase gene under the control of the HSV-TK promoter, was concomitantly transfected to normalize the transfection efficiency. After 24h, the cells were lysed and cell extracts were subjected to the "Dual-Luciferase Assay" and analyzed by a luminometer. The experiment, performed in triplicate, was repeated for a total of 10 times. Values were then normalized to the average value of the empty vector (no insert) within the same experiment to yield the vector-normalized ratio or Relative Luciferase Activity. (B) Box and whiskers plots of the relative luciferase expression from psiCHECK-2 reporter constructs transfected into HEK293T cells. Cells were transfected with the psiCHECK-2 reporter plasmid carrying the four constructs. After 24h, cell lysates were assayed for luciferase activities using the Dual-luciferase reporter assay system. Results were normalized towards the Renilla luciferase activity constitutively expressed by the psiCHECK-2 vector. The experiment, in triplicate, was repeated for a total of 10 times. Values were then normalized to the average value of the empty vector as above. (C) Box and whiskers plots of the expression index obtained from analysis of pEGFP-N1 constructs. HEK293T cells, transfected with pEGFP-N1 reporter plasmid carrying the four constructs, were analysed after 24h by flow cytometry and the expression index was calculated. The experiment was repeated 20 times, and the data underwent the statistical analysis using non parametric statistical test Kruskal-Wallis and the Dunn's multiple comparison test. Bars in the boxes represent the median values; the ends of the whiskers represent the minimum and maximum values. ***p<0.001 **p<0.01 and *p<0.05 (Kruskal-Wallis and Dunn's multiple comparison Test).

The major goal of this study was to assess whether the four possible haplotypes generated from the combination of the SNPs rs896 and rs9677 in the 3′UTR of VPAC-1 could differently influence gene expression. Accordingly, the entire 3′UTR sequence was cloned and inserted into the pGL3 expression vector which constitutively expresses the firefly luciferase. The constructs in which the four different 3′UTR regions of VPAC-1 (Table 1) had been cloned, were transiently transfected into HEK293T cells. The graph in Figure 1A, showing the results obtained through this first approach, suggests that the presence of a C at rs9677 is associated with a lower luciferase activity, compared to the T variant. It can also be noted that the C-C haplotype is associated with a maximum decrease in the luciferase activity compared to T-T haplotype, suggesting that both polymorphisms have a significant role in gene expression and their combination can determine a synergistic effect (Kruskal-Wallis p value  = 2×10^−4^; Dunn's multiple comparison post-test: C-C vs C-T p<0.01; C-C vs T-T p<0.001).

In order to validate the results obtained with the pGL3 vector and also in the attempt to obtain a more accurate normalization of transfection efficiency which might represent a bias when two different vectors are used, we repeated the experiments using the vector psiCHECK-2 which possesses both the firefly reporter expression cassette and the Renilla luciferase gene so that the first signal can be automatically normalized to the second one. The four 3′UTR haplotypes were then cloned downstream of the luciferase, and the constructs were expressed in HEK293T cells. After 24h, the transfected cells were lysed and analyzed by "Dual-Luciferase Assay" (Fig.1B). Accordingly to the previous results, the presence of a C in both rs896 and rs9677 SNPs was associated with a lower luciferase activity: Kruskal-Wallis (p value  = 0.01) and the Dunn's multiple comparison post test showed a significant difference between the C-C and the T-T haplotypes (p<0.01) thus confirming a synergistic effect of rs9677 and rs896 in modulating gene expression.

To verify the data observed in the experiments with the luciferase, the four different 3′UTR VPAC-1 haplotypes were cloned downstream of the GFP reporter gene, contained in the vector pEGFP-N1. This additional approach allowed the monitoring of the transfection efficiency as well as of the differences in the expression for each condition analyzed by flow cytometry. GFP expression was analysed at 24h after transfection (60–70% of positive cells) when the level of fluorescence was strong and no appreciable difference in the viability of cells was detectable. The experiment was repeated 20 times, and the data were analysed by the non parametric Kruskal-Wallis statistical test. The results were consistent with the luciferase assays, confirming the ranking of the different haplotypes (Fig.1C) (Kruskal-Wallis p value  = 0.006; Dunn's multiple comparison post-test: C-C vs T-T p<0.05; T-C vs T-T p<0.05).

The microRNA525-5p targets more efficiently the haplotypes carrying the rs896C sequence rather than rs896T.

**Figure 2:**
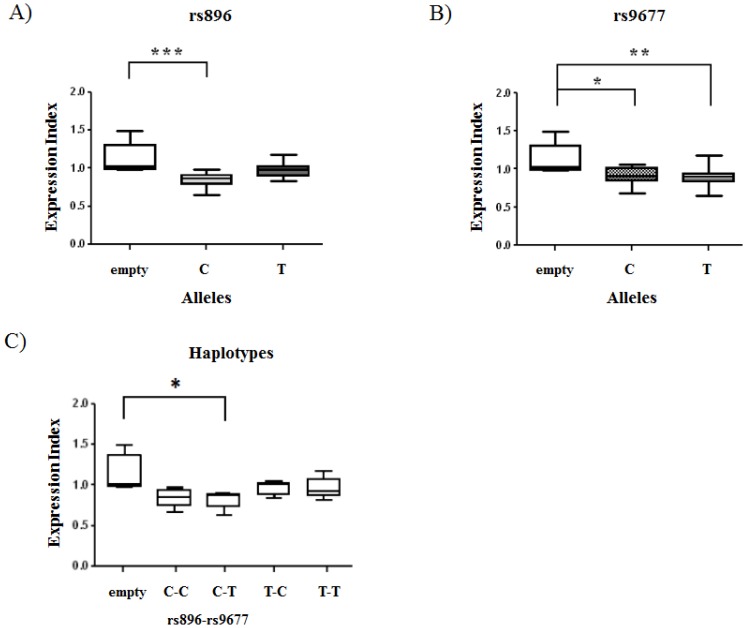
SNP rs896 affects miRNA 525-5p binding to the 3′UTR VPAC-1. Box and whiskers plots show expression index dispersion 24h after co-transfection of miRNA 525-5p or scrambled mRNAs with empty pEGFP-N1 or carrying each of the four 3′UTR VPAC-1 rs896-rs9677 haplotype constructs. The rs896 T allele is associated with a weaker miRNA 525-5p-dependent reduction in respect to the C variant (A), whereas the rs9677 allelic variants were not significantly different (B). The observed significant effect on protein modulation by miRNA 525-5p in the presence of rs896 C variant remains evident also in the haplotype analysis (C). Bars in the boxes represent the median values; the ends of the whiskers represent the minimum and maximum values. ***p<0.001 **p<0.01 *p<0.05 (Kruskal and Wallis-Dunn's multiple comparison Test).

Having assessed by independent experimental settings that the polymorphic variations at 3′UTR region of VPAC-1 influence *per se* the gene expression, we asked if the effect of microRNA 525-5p was influenced by the sequence of the two SNPs under study. The HEK293T cells carrying the four VPAC-1 3′UTR haplotypes in pEGFP-N1vector were therefore co-transfected with mimic hsa-miR-525-5p or scrambled negative-control to normalize the transfections as previously described. The experiment, performed in duplicate, was repeated 6 times and the results are summarized in Figure 2. Data confirmed that the miR525-5p did target the 3′UTR of VPAC-1, and showed that its effect was statistically different if a C or a T was present at rs896 (Kruskal-Wallis p value  = 3×10^−4^; Dunn's multiple comparison post-test: empty vs rs896C p<0.001; empty vs rs896T p = ns) (Fig.2A) but was independent from rs9677 (Kruskal-Wallis p value  = 0.002; Dunn's multiple comparison post-test: empty vs rs9677C p<0.05; empty vs rs9677T p<0.01) (Fig.2B). The reduction of the expression index in correspondence of the rs896-9677 haplotypes (Fig. 2C) confirmed the combinations C-C and C-T as more susceptible to the action of miR-525-5p, although only the evaluation of the empty vector versus the construct containing the C-T haplotype gave a statistically significant difference (Kruskal-Wallis p value  = 0.01; Dunn's multiple comparison post-test: empty vs C-T p<0.05).

## Discussion

The analysis of the entire genome in many complex disorders is beginning to reveal common patterns shared by various diseases, although the association is not necessarily with the same SNPs. Differently from the Mendelian diseases and in accordance with the tuning effect that each variant seems to have in complex diseases, it has been found that the majority of the associated variants map outside of the protein-coding regions, rather affecting regions that control the transcription (promoters, enhancers) or the RNA splicing and/or the RNA stability such as in the case of hypertension, obesity, rheumatoid arthritis and coronary artery disease [30]. Another interesting aspect of the genetic basis of complex diseases is that a relevant number of the involved genes belong to the inflammatory network. However, each variant usually accounts for a small genetic risk and all together they explain a relatively small portion of heritability for each disease. This "missing heritability" might partially be explained by gene-gene interactions and/or additive effects affecting the biochemical pathways relevant for the disease pathogenesis or by rare variations in Linkage Disequilibrium (LD) with the tagged SNPs [31], [32]. Additive effects can also result from joined action of variations mapping very close each other whose role is therefore difficult to dissect.

In this context, we have shown here that several SNPs in the 3′UTR region of VPAC1 cooperate in regulating gene expression. Previous studies from our and other groups had shown an association between the 3′UTR region of VPAC-1 gene and complex diseases [22], [24]–[27]. Furthermore, Delgado and coworkers have described an association between Rheumatoid Arthritis (RA) and some SNPs in the 3′UTR region of VPAC1 that paralleled with a decreased expression of VPAC1 during VIP-mediated signaling in PBMC of patients with RA [26].

By using different expression systems, we demonstrate here, that, depending on the rs896-rs9677 haplotype, the expression of the upstream gene was different. Moreover, rs896 turns out to act as a double cis-acting polymorphism because of its synergic effect with rs9677 in inducing a lower basal expression of the reporter genes (C-C vs T-T haplotype) and its capacity to alter the binding site of miRNA 525-5p (C vs T allele). Although the statistical significance of our results is slightly influenced by the expression system, nevertheless the rs896-rs9677 C-C haplotype always turns out to negatively affect the expression of both luciferase and GFP reporter genes. There are however some restraints in this study. Firstly, it was not possible to perform the analysis over 24 hours after transfection given the variable number of living cells in the various conditions analyzed. This imposes a limitation to identify the mechanism whereby the haplotype rs896-rs9677 acts as a cis-regulatory element of gene expression. In fact, despite its non-coding function, the 3′UTR may affect the final mRNA stability, its localization, the export from the nucleus and the translation efficiency.

During the last decade, UTRs have been shown to harbor various cis-acting elements that, in cooperation with specific binding proteins or RNAs (trans-acting elements), control the correct post-transcriptional modifications and the proteo-synthesis. The polyadenylation signal (PAS), the hexamer “AAUAAA” or, less frequently, “AUUAAA”, located approximately 10–30 nucleotides upstream of the cleavage site, were identified as highly conserved signal for the endonucleolytic cleavage at the 3' end. Therefore, variations of this sequence, e.g. SNPs, could disrupt the cleavage and polyadenylation steps resulting in pathological phenotypes, including some malignancies [33]. SNPs specifically mapping in the 3′UTR of genes may interfere with mRNA stability and translation through effects on polyadenylation and regulatory protein-mRNA and miRNA-mRNA interactions or might be responsible for the locally altered secondary structures of mRNAs, thus affecting the accessibility of binding sites for interacting trans-elements.

Many studies have recognized miRNAs as crucial regulatory molecules that may be implicated in the pathogenesis of human inflammatory and immune diseases [34]. Our data demonstrate that the T allelic variant in the SNP rs896 is not recognized as efficiently as the C variant by miRNA 525-5p, a microRNA not expressed by HEK293T cells (MicroRNA.org-Targets and Expression database and our data).

Recently, a network-based integrative analysis has highlighted the immune/microglia module as the molecular system most strongly associated with the pathophysiology of LOAD (late-onset Alzheimer's disease) [28]. One of LOAD-associated variants, rs897, is in strong linkage disequilibrium with rs896 in the 3′untranslated region of VPAC-1. Intriguingly, this variant is in close proximity to the seed binding sequence for microRNA-525-5p, and by altering the RNA secondary structure and binding kinetics, could lead to dysregulated VPAC-1 gene expression in response to disease-relevant stimuli. It would be interesting to verify whether miRNA 525-5p, which is upregulated in monocytes following an inflammatory trigger, is also expressed in brain where it could be regulated by different stimuli.

It must be considered however, that a new level of crosstalk between miRNA and 3′UTR is now emerging: miRNA binding sites can titrate away miRNAs and thereby regulate miRNAs availability and distribution, consequently leading to de-repression of miRNA target genes and even to phenotypic changes in cells, thus complicating the circuit [35].

In conclusion, our data indicate that SNP variations mapping very close each other in the 3′UTR of VPAC-1, cooperate to modify gene expression and that this effect could be further fine-tuned by miRNAs able to discriminate the allelic variants. There are indeed few examples that show how the SNP polymorphisms at the 3′UTR affect microRNA regulation [36] and rs896 seems to be one of them. Dissecting the exact mechanism by which this complex pathway is regulated, particularly in the brain, will be extremely important as this could contribute to the understanding of diseases such as Alzheimer as well as to the identification of predictive susceptibility biomarkers.
